# What Is the Vital Force?

**Published:** 1899-02

**Authors:** Stuart Close

**Affiliations:** Brooklyn, N. Y. City


### PROCEEDINGS OF THE SOCIETY OF HOMŒOPATHICIANS.

### WHAT IS THE VITAL FORCE?

By Stuart Close, M. D., Brooklyn, N. Y. City.

Two years ago I had the honor of presenting to this society a paper entitled, "What is the Law of Cure?" in which I endeavored to show the relation of the homœopathic principle to the laws of motion, and, incidentally, to show the fundamental agreement between Homœopathy and general science.

On this occasion I wish to discuss, in a somewhat similar manner and spirit, another of the great Hahnemannian doctrines, namely, the "Vital Force" or "Life Force." In doing so I shall bring to bear upon it some results of recent scientific research in the twin fields of biology and psychology, by men of recognized ability and established position in their respective fields.

I shall present, and, as far as time will allow, illustrate three theories, namely, Hahnemann's Dynamic Theory, Gates's Cellular Mentation Theory, and Hudson's Dual Mind Theory, and then endeavor to show how these theories complement and mutually elucidate each other in such parts as are not identical.

If the conclusions of our contemporary authors are not accepted in their entirety, the presentation of new thought may at least prove interesting, and may throw some light upon what has been one of the more occult phases of Homœopathy.

Hahnemann seems to use the terms "life" and "life force," or "vital force," as substantially synonymous, in many passages. In others the term "vital force" is used to express the idea of the *activity* of life as differentiated from the abstract idea of life itself. Vital force conveys the idea of life in action.

In collating the references in *The Organon* to the vital force a peculiar fact is brought out. We find that they fall naturally into two classes, which may be characterized as respectful and disrespectful.

In the first class the vital force is described and defined by the expressions "spirit like," "invisible," "known only by its effects," "omnipresent in the interior of our body," "indefatigable, active vital principle." "During the healthy condition of men this spirit-like force (autocracy), animating the material body (organism) rules supreme as *dynamis*. By it all parts are maintained wonderfully in harmonious vital process, both in feeling and functions, in order that our intelligent mind may be free to make the living, healthy, bodily medium subservient to the higher purpose of our being." (Section 9.)

"The material organism without vital force is *incapable of feeling, activity*, or *self-preservation*. This *immaterial being* (vital force) alone, animating the organism in the state of sickness and of health, imparts the faculty of feeling, and controls the functions of life." (Section 10.)

"In sickness this spirit-like, *self-acting* (*automatic*), vital force, omnipresent in the organism, is alone primarily deranged by the dynamic influence of some morbific agency inimical to life." (Section 11.)

With the body "it *constitutes a unit*, though our reason, in its processes of thought, separates this unit into two ideas, for convenience of comprehension."

It is therefore not only the basis of all the activities of the organism, but *is* the active agent itself, manifesting itself through the various organs and functions of the body, in both health and disease.

Consider the multitudinous and enormously complicated and delicate processes involved, over which the rational mind appears to exercise no control! Think only of growth, nutrition, and repair; of digestion, assimilation, and excretion; of respiration and circulation! Hahnemann is not a materialist.

He recognizes the spiritual character of life constantly. He ridicules, with biting sarcasm, those petty materialists who imagine that disease is in some way material, and endeavor to remove it from the organism by derivatives, sudorifics, cathartics, etc. He declares, with startling boldness, that "*disease is a nonentity.*" This is not to say that it is non-existent, but only that it is a *condition* and not a thing or entity. He recognizes, moreover, the vital importance and superlative value of mental symptoms in disease, based upon the intimate relation between the mind, the body, and the vital force.

Attaching so much importance to the vital force, recognizing so clearly its field of activity, and expressing his admiration of its work, as we have seen, we are surprised to find another class of expressions in which distrust, lack of respect, and almost contempt is expressed. These occur mostly in the introduction, but also in the body of *The Organon*, when he speaks of the operations of the vital force in disease. It is now this "unreasoning life-force;" "this unintelligent vital force, this blind guide;" this "crude, unreasonable, automatic vital energy," which "cannot, like a skillful surgeon, heal a wound by first intention by co-adapting its gaping edges; which does not know how to adjust and replace the divergent ends of a broken bone, notwithstanding its ability to furnish (often superabundantly) osseous matter; which cannot tie a wounded artery, but exhausts all its energy in causing a wounded person to bleed to death; which does not know how to reduce a dislocated humerus, but, on the contrary, prevents human art" (as if the vital force were not human) "from accomplishing reduction by speedily producing a swelling around the joint; which, in order to remove a splinter from the cornea, destroys the whole eye by suppuration." "Nay, this unreasonable vital force rashly receives into the body those chronic miasms (psora, syphilis, sycosis), the greatest tormentors of our earthly existence, the source of innumerable diseases, under which humanity groans for hundreds, nay, for thousands of years, and utterly unable to even palliate one of these, this same vital force is utterly incapable of removing such diseases from the organism of its own accord, but suffers them to rankle in the system until death closes the eyes of the sufferer after a long life of sorrow."

This is a formidable indictment of our vital force. How is defense to be made? Apparently there is inconsistency here. How is it that that which was so trustworthy in health is so untrustworthy in disease? How is it that that which was so expert and skillful in the performance of its duties while the person was in health is such a stupid bungler now? That which required only the simplest co-operation and was worthy of the highest confidences on the part of the rational mind in health, is now a blind guide and utterly untrustworthy. How are these statements to be reconciled?

But hear Hahnemann again:

"The vital force, capable only of acting in harmony with the physical arrangement of our organism, and without reason, insight, or reflection, was not given to us that we should regard it as the best guide in the cure of diseases." (Introduct. p. 34.)

"What man of sense would undertake to imitate nature in her endeavors of coming to the rescue? Those efforts are indeed the disease itself."

"He is in the wrong. *That noble innate power, destined to govern life in the most perfect manner during health*, equally present in all parts of the organism, in the sensitive as well as in the irritable fibre; that untiring mainspring of all normal, natural, bodily functions, was never created for the purpose of aiding itself in diseases, nor to exercise a healing art worthy of imitation. *No!* the true healing art is that *intellectual office* incumbent on the *higher human mind*, and free powers of thought, discriminating and deciding according to causes; a duty of which office is, whenever that instinctive, unconscious, and unreasoning, but automatic, energetic vital force has been thrown into discordant action by diseases, to harmonize those discordances by means of a similar pathogenetic affection of higher degree, originated by a drug homœopathically selected."

Here Hahnemann leaves the subject. If we ask how the vital force can be thrown into discordant action *by* disease, when Hahnemann has already defined that identical discordant action to *be* the disease, no answer is given. There is confusion in the use of terms. One significant point is made clear, namely, that there must be intelligent and harmonious co-operation between the "rational mind," or the "higher human mind," and the "unreasoning" vital force. Each is necessary to the other.

It seems as though Hahnemann had failed, after all, to give the vital force its true sphere and character. But we shall see, hereafter, how he was nearer the truth than appears on first consideration, or than he knew himself.

It is inherently improbable, it is absolutely unthinkable, that a blind, unintelligent force, acting automatically, or mechanically, could do any or all of the things attributed to its agency, or be to the person and its material organism what it manifestly is: things that require for their performance the exercise of choice and selection, and the adaptation of means to ends, even though the means are sometimes faulty and inefficient. There is much here requiring explanation.

It is clearly evident upon observation and reflection that *all the phenomena and operations of normal life proceed in an orderly and intelligent manner.*

Intelligence is perceptible in every functional operation in organized beings. Where and what is this intelligence? Consider the wonderful nutritive processes of digestion, assimilation, and excretion going on continuously in our bodies. We are accustomed to speak of them as being "automatically" performed, but close observation of the phenomena shows something more than mere automatism. It is possible to watch some parts of these operations under the microscope, and observers have repeatedly testified to their astonishment and fascination as they have watched protoplasmic cells moving about, selecting their course and performing their duties exactly as if endowed with intelligence. In the building up of tissues, the removal of waste products and repairs of injuries to organic structures there is constantly manifested the power of choice and selection, and the intelligent adaptation of means to ends. These are functions of mind. Pabulum is conveyed to needy parts and transformed into new tissue, and waste material carried away under the very eye of the observer. Individual cells are seen wending their devious way through tortuous channels, avoiding obstruction, never interfering with each other, until their selected destination is reached and their work accomplished. Everything moves with precision. We know that these wonderful processes are going on within our own bodies constantly, and yet we are not conscious of directly influencing or controlling them. Because we were not conscious of such control we have thought that they were independent of our mind and thought, although related in some way to us through that mysterious, intermediate, unintelligent, and unreasoning something which we call life or life force. Even the effect upon these processes of depressing mental emotions, although often observed, has had no special significance for us. The true character of the relation has escaped us.

Reason tells us that wherever work or activity proceeds systematically and intelligently there must be conscious intelligence directing it; that where there is law there must be a law giver; that behind the manifested or expressed thought there must be a thinker; that back of motion must be the moving force, and back of force *will*. Will is an attribute of mind—of intelligence. This is as true of a cell or an organ as it is of a whole organism, individual or cosmic.

The rational conscious mind of man responds to every thought presented to it by another mind. Mind responds only to mind. Proving drugs upon the healthy, according to Hahnemann, demonstrates that there is that in man which responds, in some way and under certain conditions, to the impression of every element or combination of elements in the universe. That which responds Hahnemann calls the "life force." That which makes the impression he calls "the spirit-like power concealed in drugs." He goes on to show that the only basis of this correspondence between organism and drug is their similarity of nature and character. Both are spirit-like," or "dynamic." If there is life force in the organism of man there is also life force in the organism of the plant from which the drug comes, and, by a further extension of the same argument, in the so-called "inanimate" drug substance also. All elementary substance is either living substance or capable of being transformed into living substance. Guided by the law of correspondence, which declares that like only responds to like, are we not justified in saying that all elementary substance is living substance? If the principle of life did not inhere in Silica could it respond to or impress the living organism? That in the Silica which responds to the life principle of the human organism, when they are brought into proper relations through the laws of *Similia* and *Potentiation*, is its force, or *dynamis*, as Hahnemann calls it (using the Greek work for force); and all force in the last analysis is living force. The action of Silica is not primarily upon the matter of the organism, but on the life-principle, disturbing or restoring physiological harmony, as the case may be, and this proceeds under the laws of life, acting alike in the Silica and in the organism.

Life, in the largest sense, is the sum total of all the forces in the universe. It is the grand all-inclusive primal substance, and contains within itself all the elements of being. Life in the individual is the sum total of all the elementary organic forces, and is inseparably united with the universal or cosmic life. In the individual, life is a continuous influx from the universal and infinite life, and finds its perfect manifestation through organism, in harmony with health.

Health, in the physiological sense, is a state of the organism in which, by means of a normal susceptibility to the action of the elementary forces and substances, they are continually received in proper manner and adequate amount to maintain the organism in its integrity.

So long as man is conscious of these great truths, and is master of himself through knowledge of the laws of his being, all goes well. The life force holds all in admirable harmonious sway. But let him lose this consciousness through violation of those laws and he falls from his high estate. That delicate poise and adjustment of his relations, when disturbed or lost, brings sickness, suffering, and death.

We have traced the organic processes in health and disease to life. We have seen that intelligence is involved in these processes. Where is it located, and what is the relation between life and intelligence, or mind?

In these latter days there has arisen a new investigator of physical, psychic, and mental phenomena; one who, by his fidelity to truth, by the excellence of his methods, the keenness of his philosophical insight, by his broad power of deduction and generalization, and his singular originality in devising means and apparatus for experimentation, has broadened the bounds of science in certain directions, and put forward conceptions which are new in the scientific world, although they but confirm the wisdom of inspired prophets and seers long ago recorded. He has made plain the mystical and revealed the occult.

Reference is made to the work of Professor Elmer E. Gates, formerly prominently identified with the Smithsonian Institution, of Washington, but now at the head of his own large and elaborately equipped laboratory, which is specially designed for the investigation and study of the phenomena of biology and psychology. As his researches and conclusions bear directly upon the subject under discussion I shall let him speak for himself in liberal quotations from his published papers.

From an elaborate series of observations of phenomena in his own person, under all measurable environmental conditions, and in which he recorded, four times a day for a period of two years, all measurable bodily conditions, and all mental activities, he drew "an important conclusion, namely, that our mental life, in all its acquisitive and productive capacities, is not merely directly influenced by every environmental and bodily condition, but that our mental power and processes are the results of a functional interaction between the organism of the individual human being and the larger organism of the cosmical environment. That is, mentation is the result of two factors: first, the activities of the animal organism, and, second, the activities of the cosmic organism. The relation between the two is functional. The significance of this will be seen later on when it will be shown that all physiological functionings are psychologic in their character, and that the difference between an animate and an inanimate body consists in the fact that the animate body possesses mind. The human organism is not only materially and dynamically part of the universe, but, as these experiments show, it is psychologically part of the whole."

In pursuing the course of investigations which led to these conclusions he discovered an *art of mentation* by which he was able to augment the quantity and quality of his originative work in invention and research. He practiced this art in himself as follows:

"In applying this art to myself for the promotion of my researches I first made a classified synopsis of every verified datum from all of the sciences which had any bearing upon the broad question of the study of the relation between body, mind, and environment, and then, under the previously determined bodily and environmental conditions which were found favorable to productive mentation, I passed each datum understandingly through my mind, and continued doing so, over and over again, several hours each day for some weeks, believing that by so doing I would not only render each of these data equally vivid in my apperceptive consciousness, but, that I would also, by this repeated refunctioning of certain groups of brain structures promote the growth and associative activity and elaborative processes of these structures and thus bring about superior mentative conditions more highly favorable to originative ideation and invention. Having been told, by a critical observer of my work, that there existed no proof that mental activity leaves any chemical or anatomical changes in the brain I at once proceeded to experimentally determine the facts in the case as follows:

"As has often been described in public print, I conceived the idea of giving one group of animals an excessive training in the use of some one definite mental function, and of depriving another group of animals, of same age and species, of the opportunity to use this same mental function, and then, after a year of such deprivation and training, to kill both groups of animals and contrast their brains, both chemically and microscopically, to see if in those cortical areas, where that function is located, there would be any structural differences. I found that in the seeing areas of the cortex of an animal which had been confined for one year in a darkened room there was no further appreciable development of brain cells than was to be found in an animal of the same species at the moment of birth, and that in an animal of the same species, that for the first year of its life had been trained in the extraordinary use of the seeing functions, there was a far greater number of brain cells than were to be found in an untrained animal of the same age and species that had not been deprived of light, and these brain cells were larger and more complex. By this special process of mind training, which it is the purpose of a special volume to describe, I succeeded, not merely in giving that animal more brain cells in that part of its brain than any individual of that species had ever before had, *but I also gave it more mind*, in that particular direction, than any number of that species had ever before possessed. Similar trainings with other functions corroborated these conclusions, and the experiments teach what is the functional localization in the brain of any mental faculty, and demonstrate that each conscious mental experience creates in some part of the brain a definite chemical change and structural embodiment of that experience, the refunctioning of that structure being essential to the remembering of that experience. This led to the beginning of an art of brain building for the purposes of embodying more mind.

"Inasmuch as mind creates every science and art, and constitutes the basis of all effort and of all enjoyment and suffering, it follows that to secure more mind becomes a fundamental opportunity and duty; and it follows that the animal organism is nothing more nor less than the mechanism for the manifestation of mind, and that evolution is a process of mind-embodiment—the embodiment being created by the mind's own activities. Quite recently I succeeded in showing that the same process is applicable to unicellular organisms. The simplest cell is capable of feeling a stimulus and of adapting acts to ends. *Only mind can feel and make such adaptive reactions.* A cell remembers its experiences, and *only mind can remember.* An inanimate piece of gelatine does not feel a stimulus and remember the meaning of such an experience and adapt acts to ends with reference to such memory; but a piece of protoplasm can do these things, and therefore it is animate. It follows that *life* is *mind*, and that the vital or physiologic processes are simply psychologic processes.

"When unicellular organisms are caused to perform different mental activities correspondingly different structures arise in these cells—that is, if one group of cells is caused to feel and respond to some stimulus, and if another group of the same species of cells is caused to feel and respond to a different stimulus, and if these activities are kept up in both groups for several months, there will arise structural differences between these two groups of cells which correspond to the differences between their mentative activities. Even in these physiologic units it is the mind which creates organic structure and regulates the metabolism. As is well known, all the organs of the human body are made up of cells, and each cell, as is shown by the above experiments, has its own mental life, and it is this mental functioning which constitutes its vitality. The conclusion is, that the physiological processes are explicable only as psychologic functionings.

"These experiments belong to the domain of psychologic biology, but these results have a deep medical meaning, namely, that the mind activities create and control organic structures and the metabolisms upon which all organic changes depend. An animal is a mind organism. The cells out of which an animal is built are mind organisms, and the duties of each cell are duties which require mind for their performance. A cell cannot perform its functions in the animal economy except in so far as it is capable of *feeling* stimuli and in so far as it is capable of *adapting acts to ends.* To change the mental characteristics of a cell is to alter its physiologic meaning in the animal economy."

"If mind activities create chemical and anatomical changes in the cells and tissues of the animal body it follows that all physiologic processes of health and disease are psychologic processes."

From the effects of environmental conditions upon the human individual, and from certain rhythmic phenomena that are synchronous in man and the earth, he goes on to prove that the mind of the human being is functionally connected with the cosmic whole, and that the human being is an organ in a larger organism.

It is a striking corroboration by the methods of science of the grand truths of the solidarity of the race, of the fatherhood of God and the brotherhood of man, of the unity in Christ of the children of God, taught by Jesus in the parable of the Branch and the Vine, of the teaching of Swedenborg in the figure of the Grand Man, and in many other forms and figures. The idea is as old as the truth itself, but Prof. Gates has demonstrated it by the methods, and clothed it in the language of science.

I have not time to refer to or quote further from the very elaborate experiments upon which these conclusions are based. Prof. Gates is his own severest critic, and his conclusions are entitled to respect. Reviewing his work he says:

"Suffice it to say *the evidence is complete* which demonstrates that every mental activity creates a definite chemical change and a definite anatomical structure in the animal which exercises that mental activity, and that this is the *modus operandi* of animal growth and evolution, and that by this method more mind can be embodied *ad libitum.*

"*The evidence is complete* which shows that every mentation also produces a definite effect upon the environment of the animal which does the mentating.

"Action and reaction are equal. Force cannot come from nothing. Mentation is a mode of energy, and the organism of the animal cannot create the energy of life out of nothing, but must receive it from the great reservoir. *But the conclusion that every mentation affects the environment is based upon direct testimony and quantitative measurement.* Vary the mental activities of an unicellular organism and you will vary its structures, and the same is true of a multicellular dog or man. Mind underlies organic phenomena, and life is mind, and mind activity is the cause of evolution, and mind embodiment is the goal."

He reiterates: "Mind creates every science and art, and therefore the science of mind—psychology—is the science of the sciences; and therefore the art of using the mind and the art of getting more mind—psychurgy—is the art of arts. Mind is life. Life is not something different from mind. The life of a cell is its mind. The activities of a cell are psychologic activities.

"Life and vitality and physiologic processes are solely mental processes."

We now have an answer to our question, "What is Life?" We see why the operations of life in vital processes proceed intelligently. Thus far we have studied the matter from the biological standpoint, in what may be called normal relations. When we come to study the organism under abnormal conditions, as in disease, difficulties arise. It seems as if vital processes should proceed as intelligently in disease as in health, but judging from the effects of disease Hahnemann was moved to call the life force blind, unintelligent, unreasoning, helpless, or worse, and apparently he was right. Prof. Gates says "Pathology is abnormal *cellular* mentation." In this sentence is the key to the problem, as it is also in Hahnemann's phrases, "*rational* mind" and "*higher* human mind" already referred to. These phrases imply the existence of a *lower* human mind. For the further elucidation of this matter I shall appeal to another class of investigators, and specifically to one author, Thompson Jay Hudson, in whose book, *The Law of Psychic Phenomena*, the first satisfactory attempt is made to harmonize various more or less conflicting theories, and to propound a working hypothesis for the study and investigation of the vast mass of psychic phenomena.

Mr. Hudson's book is quite in harmony with the teaching of Prof. Wm. James, of Harvard, the leading American psychologist, and we shall see how it applies to Prof. Gates's position.

It is quite superfluous to say that the human mind is a very wonderful thing. It is wonderful enough in its ordinary more or less normal operations, but when we begin to investigate the phenomena of telepathy, of somnambulism or trance, of hypnotism, of mesmerism, of spiritism, of the various forms of mental or metaphysical healing, etc., to say nothing of certain forms of insanity and other diseases, we begin to sympathize with the simple rustics in Goldsmith's "Deserted Village," as they listened to the village schoolmaster engaged in an argument with the parson:

> "Amazed, the gazing rustics ranged round—
> And still they gazed, and still the wonder grew,
> That one small head could carry all he knew."

A subject in hypnotic trance, for example, brings up from his inner consciousness so many things he didn't know he knew, and does so many impossible things that we rub our eyes in amazement.

There have been many theories about all this, but no satisfactory working hypothesis until Mr. Hudson presented his, and his book has created an epoch in psychological studies.

During trance, artificially produced, and sometimes during sleep, or natural trance, and in certain febrile conditions the mind may compose beautiful poems (Coleridge thus produced his most exquisite poem—"Kubla Khan"); may deliver learned disquisitions; pronounce orations; solve intricate mathematical problems, as Sir John Herschel did; complete valuable inventions, suspended in despair during waking hours, as Edison has repeatedly done; compose music; write sermons, as Spurgeon once did; recall experiences long forgotten, and do many other remarkable things, all the time being perfectly oblivious to the external world with every objective sense in abeyance, and of which there is not the slightest recollection after waking.

It is not necessary, nor is there time to give illustrations of these peculiar powers of the mind as brought out in the various lines of thought already referred to. They are doubtless familiar to you all. The public print has been full of them for years. I will therefore only point out some significant facts in regard to these phenomena, bring forward the hypothesis which explains them, and then show its application to the subject immediately in hand.

I quote from Hudson: "It is well known to hypnotists that when an idea is suggested to a subject, no matter of how trivial a character, he will persist in following that idea to its ultimate conclusion, or until the operator releases him from the impression. For instance if a hypnotist suggests to one of his subjects that his back itches, to another that his nose bleeds, to another that he is a marble statue, to another that he is an animal, etc., each one will follow out the line of his particular impression, regardless of the presence of others, and totally oblivious to all his surroundings which do not pertain to his idea; and he will persist in doing so until the impression is removed by the same power by which it was created. The same principle prevails when a thought is suggested and the subject is invited to deliver a discourse thereon. He will accept the suggestion as his major premise, and whatever there is within the range of his own knowledge or experience, whatever he has seen, heard, or read, which confirms that idea, he has at his command and effectually uses it, but is totally oblivious to all facts or ideas which do not confirm and are not in accord with the one central idea. It is obvious that inductive reasoning, under such conditions, is out of the question."

This is characteristic of all hypnotic conditions, and Hudson goes on to point out the factors upon which he bases his hypothesis. The principal one is that "*hypnotic subjects are constantly amenable to the power of suggestion; that suggestion is the all-potent factor in the production of all hypnotic phenomena.*" He then lays down three propositions, drawn from exhaustive studies of the phenomena of hypnotism, telepathy, mental healing, etc. These propositions are as follows:

First, that "man has, or appears to have, *two minds*, each endowed with separate and distinct attributes and powers; each capable, under certain conditions, of independent action. It should be clearly understood at the outset that for the purpose of arriving at a correct conclusion it is a matter of indifference whether we consider that man is endowed with two distinct minds, or that his one mind possesses certain attributes and powers under some conditions and certain other attributes and powers under other conditions. It is sufficient to know that everything happens just as though he were endowed with a dual mental organization."

He assumes this to be true, and designates one as the *objective*, and the other as the *subjective* mind. Other authors use the terms *conscious* and *subconscious* mind, or self, and still others speak of the *subliminal* mind, or self.

His second proposition is that *the subjective mind is constantly amenable to control by suggestion.*

The third or subsidiary proposition is, that *the subjective mind is incapable of inductive reasoning.* He shows that the broad idea of the duality of man's mind is very old, but that it has always been indefinite, and that no successful attempt has ever before been made to define clearly the nature of the two elements. He says:

"In general terms the difference between man's two minds may be stated as follows:

"The objective mind takes cognizance of the objective world. Its media of observation are the five physical senses. It is the outgrowth of man's physical necessities. It is his guide in his struggle with his physical environment. *Its highest function is reasoning.*

"The subjective mind takes cognizance of its environment by means independent of the physical senses. It perceives by intuition. It is the seat of the emotions, and the storehouse of memory. It performs its highest functions when the objective senses are in abeyance. In a word, it is the intelligence which makes itself manifest in a hypnotic subject when he is in a state of somnambulism."

In this state many of the most wonderful feats of the subjective mind are performed. It sees without the use of the natural organs of vision, and in this, as in many other grades or degrees of the hypnotic state, it can be made, apparently, to leave the body, and travel to distant lands and bring back intelligence, oftentimes of the most exact and truthful character. It also has the power to read the thoughts of others, even to the minutest details; to read the contents of sealed envelopes, and of closed books. In short it is the subjective mind which possesses what is popularly known as clairvoyant power, and the ability to apprehend the thoughts of others without the aid of the ordinary objective means of communication.

Hudson's opinion is that the objective mind is merely the function of the physical brain, while the subjective mind appears to be a distinct entity, possessing independent powers and functions, having a mental organization of its own, and being capable of maintaining an existence independently of the body. In other words, he believes it to be the soul. He does not attempt to localize it any more definitely than this. Special emphasis is laid upon the unqualified susceptibility of the subjective mind to suggestion. "The subjective mind accepts without hesitation or doubt, every statement made to it, no matter how absurd or incongruous or contrary to the experience of the individual. If a subject is told he is a dog he will instantly accept the suggestion, and, to the limit of physical possibility, act the part suggested. He may be thrown into a state of intoxication by being made to drink a glass of water under the impression that it is brandy. If told that he is in a high fever, his pulse will become rapid, his face flushed, and his temperature increased." These facts have been demonstrated thousands of times.

It is also true that "the subjective mind of an individual is as amenable to the control of his own objective mind as to the objective mind of another," and in this we have the key to the cause and cure of certain forms of disease by mental means.

"The objective mind is capable of reasoning by all methods, —inductive and deductive, analytic and synthetic."

"The subjective mind is incapable of inductive reasoning, while the individual is in the state of hypnotism or trance." "The feats of the subjective mind have caused amazement for ages, but it has never been noticed heretofore that its reasoning is always deductive or syllogistic. It never classifies a series of known facts, and reasons from them to general principles; but given a general principle to start with it will reason from that down to all legitimate inferences with a marvellous cogency and power."

There is good ground for believing that the memory of the subjective mind is perfect; that no experience through which the individual passes, nothing that he has ever perceived through any of his senses is ever lost. Many illustrations of this could be given if time permitted; of extraordinary feats of memory during illness—of a forgotten language recovered; of whole pages of Greek and Hebrew remembered and recited by an illiterate servant girl, who, years before had worked in the house of a preacher who was in the habit of reading aloud passages from Greek and Hebrew authors, which her ear casually heard while she was working about the room, etc. Suffice it to say that Mr. Hudson's book of four hundred pages is devoted to the application of this hypothesis to the explanation of a vast number of strange and mysterious things, and that it does explain them satisfactorily.

I have already quoted his opinion that the subjective mind is a separate entity, independent of the physical organism. In another place he says, "Subjective memory appears to be an inherent power, and free from anatomical relations, *or at least it does not appear to depend upon the healthy condition of the brain* for its power of manifestation."

At this point, it seems to me, the theories of Hahnemann, of Prof. Gates, and Mr. Hudson meet and complement each other. Something was lacking in each which the others supply, and a fair consideration of all greatly elucidates the subject we are discussing. Hahnemann's "life force" corresponds to Prof. Gates's "cellular mind," which is nothing more nor less than Hudson's "subjective mind." Prof. Gates definitely locates Hudson's subjective mind and shows how it does its work in bringing about structural changes. A little reflection will show why Hahnemann's life force is "blind," "unreasoning," etc., and why it is not to be trusted in disease. It is subject to every evil suggestion, conveyed from the objective mind of its own or other individualities, and it can only reason deductively. Every mental functioning works tissue change, and the physical evidences of disease are the result.

Hahnemann's "rational mind," and "higher human mind" correspond to Hudson's "objective mind," the highest function of which is to reason, and which is able to reason inductively as well as deductively, analytically as well as synthetically.

Hahnemann's vital force was not, as he thought, unintelligent, unreasoning, and blind, neither is it automatic in the ordinary sense of the word. It reasons correctly, but from a false premise. Working in harmony with the "rational" or objective mind, during health it manifests the highest intelligence and most beautiful reasoning powers, as Hahnemann partly recognized. It is only when it is divorced from the objective mind and subject to evil suggestion that it is at fault. Even then it is true and consistent, and even admirable, though wasting its energy in reasoning on a false premise, as when it suppurates an eye away in the attempt to remove a splinter from the cornea. If the "rational mind" would first remove the splinter, this same subjective mind, this same "blind and unintelligent force" would immediately and effectually heal the wound, *and by precisely the same process that, carried too far, becomes suppuration.* The same is true in the case of the broken bone and other illustrations used by Hahnemann, already quoted, and easily explained. Prof. Gates' studies of pathology, or as he calls it, "abnormal cellular mentation," have not yet reached a point where he has discovered the laws under which this morbid mentation proceeds. Mr. Hudson has done a large part of that work for him, as has been shown.

The attention of the psychologists has all been centered upon the performance of the subjective mind during the *abnormal* condition of the spontaneous or induced hypnotism. They have noticed the wonderful modification of organic and mental functions during this state and have correctly explained them. But it has occurred to nobody that the power which intelligently presides over those normal functions of the human organism which go on quite independent of the objective mind, such as digestion, nutrition, and excretion, is identical with that power which operates in the abnormal hypnotic condition, namely, the subjective mind. Prof. Gates's experiments demonstrate it although apparently he has not yet made this deduction.

No one seems to have seen the stupendous possibilities of the Hahnemannian "proving" as a method and instrument in biological and psychological research. No other means is so potent to bring into view every function of the economy—mental and physical.

It is only when, in the slow progress of science along the higher lines, we find the most advanced of the workers developing and using methods which are essentially but modifications of that devised and used by Hahnemann nearly a century ago, that we realize how great is our inheritance from that inspired man. The beauty and utility of his method of sounding the depths of the human mind and body consisted in its directness, simplicity, and naturalness. Men of to-day engaged in analogous or related lines of investigation are trying to accomplish similar results by methods which, though similar, are vastly more complicated and indirect, not knowing that an instrument, formed and perfect, is lying ready at their hand.

Prof. Gates may well devote some time to a careful study of Hahnemann's *Organon*, with special attention to the method and principles of drug proving. If he puts it to practical use Hahnemann's debt to him for elucidating the vital force will be more than offset, for Prof. Gates thinks he is on the eve of discovery of the fundamental law of cure, in addition to all his other great discoveries. If, perchance, his experiments should really lead him aright, and he should discover the law of cure, may it be my happy privilege to point out to him that he has been anticipated by Hahnemann nearly a hundred years. And thus shall we prove the truth of the doctrine that we are all not only debtors to, but *members* of one another, and that our perfection as individuals, and as a cosmic whole, is the goal of human effort.

